# Efficient Implementation of Cerebellar Purkinje Cell With the CORDIC Algorithm on LaCSNN

**DOI:** 10.3389/fnins.2019.01078

**Published:** 2019-10-15

**Authors:** Xinyu Hao, Shuangming Yang, Jiang Wang, Bin Deng, Xile Wei, Guosheng Yi

**Affiliations:** School of Electrical and Information Engineering, Tianjin University, Tianjin, China

**Keywords:** Purkinje, multiplier-less, coordinate rotation digital computer (CORDIC), field-programmable gate array (FPGA), digital implementation

## Abstract

Purkinje cell is an important neuron for the cerebellar information processing. In this work, we present an efficient implementation of a cerebellar Purkinje model using the Coordinate Rotation Digital Computer (CORDIC) algorithm and implement it on a Large-Scale Conductance-Based Spiking Neural Networks (LaCSNN) system with cost-efficient multiplier-less methods, which are more suitable for large-scale neural networks. The CORDIC-based Purkinje model has been compared with the original model in terms of the voltage activities, dynamic mechanisms, precision, and hardware resource utilization. The results show that the CORDIC-based Purkinje model can reproduce the same biological activities and dynamical mechanisms as the original model with slight deviation. In the aspect of the hardware implementation, it can use only logic resources, so it provides an efficient way for maximizing the FPGA resource utilization, thereby expanding the scale of neural networks that can be implemented on FPGAs.

## Introduction

The cerebellum is a very important part of the human brain and associated with many important functions with a large number of incoming and outgoing connections between the brain, brainstem, and spinal cord. These functions are not only relevant to motor control including error correction (Doya, 2000; Llinas, 2009), tracking movements (Paulin, 1993; Miall et al., 2000), and coordinated movements (Thach et al., 1992; Heck et al., 2007) but also relevant to many non-motor functions such as linguistic prediction, word generation, emotional control, and so on (Leiner et al., 1993; Schmahmann and Caplan, 2006; Pleger and Timmann, 2018). Purkinje cells (PCs) make up the middle layer of the cerebellum, Purkinje layer, which is responsible for receiving information from the cerebellar granule cell (GC) synapses through parallel fibers (PF) and climbing fibers (CF) in brainstem. In addition to being all the constituent cells of the cerebellar Purkinje layer, PCs also directly connect to deep cerebellar nuclei cells, which are the main output cells of cerebellum. So, it is obvious that PCs play the most important role in the information processing of the cerebellum. Besides, PCs are responsible for cerebellar motor learning (Gilbert and Thach, 1977) with the information stored in the synapses with granule cells. The information is presented as the variation of synaptic strength according to the error signals carried by CFs through spike timing-dependent plasticity (STDP), which consists of long-term potential (LTP) and long-term depression (LTD) (Ito and Kano, 1982; Han et al., 2000; Medina et al., 2000). This learning mechanism can be obviously observed in classical eyeblink conditioning experiments (Bao et al., 2002) and cerebellar vestibulo-ocular reflex (VOR) (Blazquez et al., 2003; Masuda and Amari, 2008), which are mainly caused by the function of PCs.

There are two calculation modes for simulation spiking neurons or spiking neural networks, serial computing mode, and the parallel computing mode (Yang S. M. et al., 2019). The serial computing mode is mainly based on some computer simulation software that is incompatible with the parallel computing features of real neural systems. In order to achieve these in a more biological way, more and more neuroscientists prefer to implement neurons and neural networks with parallel computing mode. Analog very Large-Scale Integration (VLSI), Graphics Processing Unit (GPU), and Field Programmable Gate Array (FPGA) are the three most used platforms with parallel computing capacity. Analog VLSI is an efficient analog-based method for hardware implementation of spiking neurons and neural networks because it can realize the non-linear function directly (Han, 2005; Hsieh and Tang, 2012). However, it cannot be flexibly changed once formed, so it is more suitable for well-defined circuits. In addition, its high cost and long development cycle also limit the application range. GPU provides a digital implementation method for spiking neurons and neural networks with its powerful parallel calculation ability and many researches have been carried on GPUs (Igarashi et al., 2011; Yamazaki and Igarashi, 2013). However, the kernel-launch method used on GPU and the limited bandwidths are obstacles for dealing with a lot of data. Compared to the two methods above, FPGA has many advantages for realizing the neural circuits. On one hand, the flexible reconfigurability and parallel computing architecture can perfectly meet the requirements for exploring characteristics of not only spiking neurons but also the large-scale spiking neural networks; on the other hand, its low area and power consumption also make it popular in neurosciences (Yang et al., 2017, 2018a). In this work, the neuron is implemented on the Large-Scale Conductance-Based Spiking Neural Networks (LaCSNN) system first proposed by Yang S. et al. (2019). The system consists of six Altera EP3SL340 FPGAs and is designed to simulate large-scale spiking neural networks with digital neuromorphic architecture. Its powerful storage capacity, high calculation speed, and sufficient resources make it an effective tool for neuroscience researches.

Although the advantages of FPGA are very prominent, the disadvantages are also distinct. Most of the resources on FPGA are logic resources; the lack of memory and multiplier resources often limits the scale when implementing neural networks. As a kind of digital systems, it is difficult to implement the non-linear functions directly. To solve these problems, many methods have been proposed. One of the most frequently used methods is to store the function values in a storage area with continuous address space in advance, which is called look up table (LUT) realization. When used, the function value can be obtained by addressing. This method is very easy but costs much memory resources. Besides, the use of LUTs increases the duration of reconstruction when changing model parameters. Another method, Taylor series approximation, is to replace the non-linear function in the neighborhood with an *n*-order polynomial approximation for a certain error. This method can make a trade-off between LUT resources and multiplier resources with different approximation order, but it still needs these resources (Lee and Burgess, 2003). The piece-wise linear (PWL) approximation (Julian et al., 1999) is a more efficient method to solve these problems but there are two main cons: one is there will be unavoidable error due to the use of several linear segments; the other is that it needs to recalculate when the non-linear function changes. So, in this work, we propose a non-multiplier and non-LUT method with the CORDIC algorithm for implementing the cerebellar Purkinje model on FPGA.

One of the main reasons for implementing single neurons with optimization algorithms on FPGA is to lay a foundation for realizing large-scale spiking neural networks. Many researches have been carried out in recent years. Yang et al. (2018b) propose a series of techniques for implementing a conductance-based neuron model that is beneficial for building large-scale neural networks. Soleimani et al. (2012) implement a classic Izhikevich model using PWL method to prove that the method can simplify the hardware implementation with showing similar dynamic behaviors. Ambroise et al. (2013) also implement an Izhikevich model on FPGA, but it is mainly to propose an architecture to reproduce a neural network with only one computation core (one neuron) based on one multiplier. Bonabi et al. (2012) implement a Hodgkin–Huxley (H–H) single neuron with the CORDIC algorithm and some LUTs that show high precision with more compact used logic.

There are also many researches about implementing the CORDIC algorithm on FPGA. Valls et al. (2002) evaluate some methods for the CORDIC algorithm and realize a variable precision method using conventional arithmetic on FPGA. Liu et al. (2014) implement a modified CORDIC algorithm that reduces the utilization of ROM resources and power consumption. Garcia et al. (2006) realize a pipelined CORDIC architecture with solution for overflow and quadrant correction and successfully generating sine and cosine waves. Muñoz et al. (2010) propose a floating-point CORDIC FPGA implementation for calculating transcendental functions. The FPGA implementation of the CORDIC algorithm can give full play to the advantages of FPGA and utilize hardware resources to realize an optimization scheme combining hardware and algorithm. The pipelined computational structure of FPGA can also enhance the real-time performance of the CORDIC algorithm, minimizing the computational delay due to the iterative operations. Therefore, the CORDIC algorithm can be widely applied to real-time high-quality signal processing with high-performance requirements.

The remaining parts of this work are arranged as follows. In section Neuron Model, the original model and modified CORDIC model of cerebellar PC are presented. The CORDIC algorithm used is also introduced in this section. Section Hardware Implementation Based on LaCSNN describes the details of hardware implementation. The results of software simulation and hardware simulation are shown in section Results. We also compare and analyze the result between the original model and the CORDIC model with various evaluation indicators for both the two simulations. The behaviors of a network with this neuron are also presented. section Discussion illustrates the discussion and conclusion for this work.

## Neuron Model

### Original Purkinje Model

During the exploration of PCs, many mathematic models have been built for different research interests (De Schutter and Bower, 1994a,b; Khaliq et al., 2003). Many models are either too detailed to form a large-scale neural network or too simple to have many basic biological characteristics. For the starting point of our implementation, which is to propose a method for simplifying a single neuron model with relatively high biological plausibility and make contributions to build large-scale networks on FPGA, we choose an H–H (Hodgkin and Huxley, 1952)-based model proposed by Miyasho et al. (2001) and Middleton et al. (2008), which consists of 32 ionic channels and simplified by Kramer et al. (2008) to 5. The membrane potential is shown as follows:

(1) $$\begin{array}{l} C\frac{dV}{dt}=-g_{k}n^{4}\left(V-E_{k}\right)-g_{Na}m_{\infty }{\left(V\right)}^{3}h\left(V-E_{Na}\right)\\ \;-g_{Ca}c^{2}\left(V-E_{Ca}\right)-g_{M}\;M(V-E_{M}\;)\\ \;-g_{L}\;(V-E_{L})-I \end{array}$$

where *V* represents the membrane potential, *C* represents the membrane capacitance, and *g*_*i*_ and *E*_*i*_ (*i ϵ* {*k, Na, Ca, M, L*}) are the maximum ionic conductance and reversal potentials for different ion channels, respectively. There are five ionic currents and an external stimulus current *I* in this model: a potassium current $I_{k}=g_{k}n^{4}\left(V-E_{k}\right)$, a sodium current $I_{Na}=g_{Na}m_{\infty }{\left(V\right)}^{3}h\left(V-E_{Na}\right)$, a calcium current $I_{Ca}=g_{Ca}c^{2}\left(V-E_{Ca}\right)$, an M-current *I*_*M*_ = *g*_*M*_*M*(*V* − *E*_*M*_), and a leak current *I*_*L*_ = *g*_*L*_(*V* − *E*_*L*_). *n, m, h, c*, and *M* are gating variables for different ionic currents and the dynamics are described as follows:

(2) $$\begin{array}{r} x_{\infty }=\frac{\alpha_{x}}{\alpha_{x}+\beta_{x}},\;\tau_{x}=\frac{1}{\alpha_{x}+\beta_{x}} \end{array}$$

(3) $$\begin{array}{r} \frac{dx}{dt}=\frac{x_{\infty }-x}{\tau_{x}} \end{array}$$

*x*_∞_ is the state variable, τ_*x*_ is the time constant for *xϵ* {*n, m, h, c, M*}, α_*x*_, and β_*x*_ are relevant functions, and all of these are functions of membrane potential *V*. The detailed parameter values and the description for ionic currents dynamics are provided in Table 1.

**Table 1:** Conductance parameters of cerebellar Purkinje cell.

**Current type**	***g*_*i*_**	***E*_*i*_**	***x*_∞_ (α_*x*_)**	**τ_*x*_ (β_*x*_)**
Potassium	10	−95	1/{1+exp[−(*V* + 29.5)/10]}	0.25+4.375exp[(*V* + 10)/10], *V* ≤ −10
				0.25 + 4.375exp[−(*V* + 10)/10], *V*>−10
Sodium	125	50	1/{1 + exp[(*V* + 59.4)/10.7]}	
			*m*_∞_ = 1/{1 + exp[−(*V* + 34.5)/10]}	0.15 + 1.15/{1 + exp[(*V* + 33.5)/15]}
Calcium	1	125	1.6/{1 + exp[−0.072(*V*−5)]}	0.02(*V* + 8.9)/{exp[(*V* + 8.9)/5]−1}
M	0.75	−95	0.02/{1 + exp[−(*V* + 20)/5]}	0.01exp[−(*V* + 43)/18]
Leak	2	−70	–	–

### CORDIC-Based Purkinje Mode

In order to make the implementation more suitable for building large-scale neural network and improve the calculation speed, we modify the original Purkinje model to save memory and multiplier resources with the CORDIC algorithm and introduce as follows.

The CORDIC algorithm is originally developed in Volder (1959) as an algorithm for calculating trigonometric and hyperbolic functions and first used in navigation systems. Then, a unified CORDIC algorithm is proposed in Walther (1971). By introducing a coordinate system parameter *m*, the circular rotation, hyperbolic rotation, and linear rotation are unified into the same CORDIC iterative equations, which provide a premise for the multifunction of the same hardware implementation. The essence of the CORDIC algorithm is to approximate a certain rotation angle by using a set of constant angle bases. It is possible to accurately calculate many non-linear functions by using vector repeated rotation. Its iterative equation is as follows:

(4) $$\{\begin{array}{ll} {\text{X}}_{\text{i+1}}\text{=} & X_{i}-{\text{mq}}_{\text{i}}{\text{Y}}_{\text{i}}\cdot {\text{2}}^{-\text{i}}\\ {\text{Y}}_{\text{i+1}}\text{=} & {\text{X}}_{\text{i}}{\text{+q}}_{\text{i}}{\text{X}}_{\text{i}}\cdot {\text{2}}^{-\text{i}}\\ {\text{Z}}_{\text{i+1}}\text{=} & {\text{Z}}_{\text{i}}{-\text{q}}_{\text{i}}\cdot \theta_{\text{i}} \end{array}$$

where *X*_*i*_ and *Y*_*i*_ are the value before rotation, *X*_*i*+1_ and *Y*_*i*+1_ are the value after rotation, *q*_*i*_ is the direction of rotation, θ_*i*_ and the relationship between *m* value and rotation mode are both described in Equation (5).

(5) $$\theta_{i}=\{\begin{array}{lll} \tanh^{-1}\left(2^{-i}\right), & m=-1, & \text{hyperbolic rotation}\\ 2^{-i}, & m=0, & \text{linear rotation}\\ \arctan(2^{-i}), & m=1, & \text{circular rotation} \end{array}$$

The exponential operations and divisions used in this paper are calculated in the hyperbolic rotation and linear rotation modes, respectively. The division can be easily gotten with $\theta_{i}=2^{n-i}\left(\theta_{i}{=2}^{i-n}\right),\;$where *n* determines the calculation range. As for the exponential operations, since through hyperbolic rotation we can only obtain the values of *coshθ* and sinhθ, *e*^θ^ needs to be calculated with the basic relationship between hyperbolic functions sinhθ+*coshθ* = *e*^θ^. According to Equation (5), we can know that the convergence domain is limited by tanh^−1^(2^−*i*^). In detail, the maximum value it can be calculated is determined by the sum of all the angles, which is approximately equal to 1.1182. It is obvious that it cannot meet the calculation requirements of this model. So, before calculating, the input variable needs to be preprocessed to expand the convergence domain. Suppose the input variable is θ, it can be divided into integer part *A* and fractional part *b* after being divided by ln2 just as Equation (6) and the exponential operation will be *e*^θ^ = 2^*A*^·*e*^*bln*2^.

(6) $$\begin{array}{r} \frac{\theta }{ln2}=A+b \end{array}$$

There are two reasons for choosing *ln*2, one is the exponential operation of integer part can be transmitted to power of 2 directly, which can be easily implemented by shifting; the other one is that *b*·*ln*2 is smaller than 1.1182, which is just within the convergence domain. With this method, we can perform exponential operations in any range. After careful consideration, in the case of ensuring high precision and minimizing resource consumption, the iterations in this work are chosen as: 10 for exponential operations and 12 for divisions with *n* = 2 for θ_*i*_.

## Hardware Implementation Based on LaCSNN

To the best of our knowledge, there are no works on FPGA implementation for cerebellar PC model based on H–H form. The detailed implementation method is described in the following.

In order to be implemented on a digital system, the differential equations of the Purkinje model should be solved with the Euler method. The Euler method is suitable for hardware implementation with its easy operation and adequate precision. The discretization results with a mathematical finite-difference method are shown as follows, Equation (7) is for membrane potential and Equation (8) shows the results of other variables:

(7) $$\begin{array}{l} \text{V}\left[\text{k+1}\right]\text{=V}\left[\text{k}\right]\\ +\{\begin{array}{l} g_{\text{k}}n^{4}\left(\text{V}\left[\text{k}\right]{-\text{E}}_{\text{k}}\right)-{\text{g}}_{\text{Na}}{\text{m}}_{\infty }{}^{\text{3}}\text{h}\left(\text{V}\left[\text{k}\right]{-\text{E}}_{\text{Na}}\right)\\ -{\text{g}}_{\text{Ca}}{\text{c}}^{2}\left(\text{V}\left[\text{k}\right]{-\text{E}}_{\text{Ca}}\right)\\ -{\text{g}}_{\text{M}}\text{M}\left(\text{V}[\text{k}]{-\text{E}}_{\text{M}}\right){-\text{g}}_{\text{L}}\left(\text{V}\left[\text{k}\right]{-\text{E}}_{\text{L}}\right)-\text{I} \end{array}\;{\}}^{*}(\frac{\Delta \text{t}}{\text{C}}) \end{array}$$

(8) $$\{\begin{array}{ll} \text{h}\left[\text{k+1}\right] & =\text{h}\left[\text{k}\right]\text{+}{\frac{{\text{h}}_{\infty -}\text{h}[\text{k}]}{\tau_{\text{h}}}}^{*}\Delta \text{t}\\ \text{n}\left[\text{k+1}\right] & =\text{n}\left[\text{k}\right]\text{+}{\frac{{\text{n}}_{\infty -}\text{n}[\text{k}]}{\tau_{\text{n}}}}^{*}\Delta \text{t}\\ \text{c}\left[\text{k+1}\right] & =\text{c}\left[\text{k}\right]\text{+}{\left(\alpha_{\text{c}}^{*}\left(1-\text{c}\left[\text{k}\right]\right)-\beta_{\text{c}}^{*}\left[\text{k}\right]\right)}^{*}\Delta \text{t}\\ \text{M}\left[\text{k+1}\right] & =\text{M}\left[\text{k}\right]\text{+}{\left(\alpha_{\text{M}}^{*}\left(1-\text{M}\left[\text{k}\right]\right)-\beta_{\text{M}}^{*}\text{M}\left[\text{k}\right]\right)}^{*}\Delta \text{t} \end{array}$$

where *k* is the iterations and Δ*t* is the time step for the Euler method. Generally speaking, the precision is inversely proportional to the value of the time step, and in this work, the time step is set to be 0.004 ms, which is the same in Traub et al. (2003).

Since floating-point operations take up a lot of resources and require a long calculation time, the FPGA implementation usually uses fixed-point calculations under the premise of meeting the computing needs. The bit width of fixed-point calculation is another important factor affecting precision or even implementation result besides the time step. The selection of bit width can be divided into an integer part and a fractional part, which can be estimated according to the software simulation results. For example, the range of *V* in this work is −60 to 40 mV, so the integer part should be 7 at least for 2^7^/2 > |−60|. If we need the precision to be 0.001, the bit width of the fractional part that directly determines it should be 10. In the calculation process of this work, the range of most variables is from −100 to 100, so the bit width of the integer part for most logical operation modules is 8. It should be pointed out that one of the variables reached 8,000 in the process of calculating *h*_∞_, so the bit width of the integer part for related logical operation modules is 14. In order to guarantee the precision of spiking and dynamics, the bit width of the fractional part is chosen to be 15.

**Figure 1:**
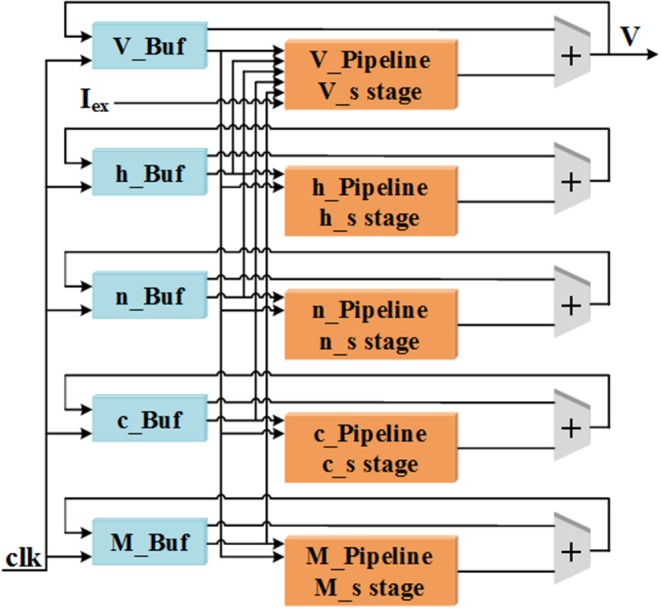
The diagram of pipeline structure for the five variables used in cerebellar Purkinje model.

In this work, all of the variables including *V, n, h, c*, and *M* in Equations (7) and (8) are designed to be realized with pipeline structures. The overall pipeline schematic is shown in Figure 1. There are two parts for one pipeline structure, the “Pipeline” includes all the calculations in Equations (7) and (8) and the “Buf” consists of a certain number of buffers to store the calculation results for each variable. This implementation method can improve the throughout and calculation efficiency of the LaSCNN system.

**Figure 2:**
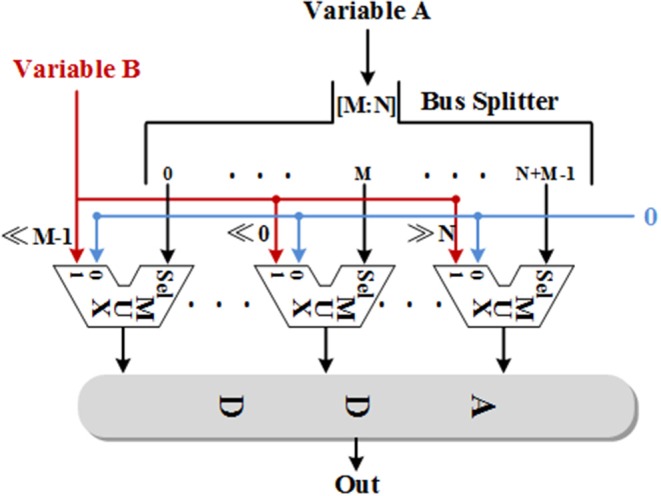
The schematic diagram of the functional shift multiplier (FSM).

It is well-known that one of the factors limiting the size of the network implemented on the FPGA is the limited multiplier and memory resources. Due to most of the resources on FPGA is logic resources, all the multiplication, division, and exponential operations are replaced by adders and shifters in this work. The division and exponential operations are implemented with the CORDIC method as described in section Neuron Model and the multiplications are implemented through two methods, which are more efficient than the CORDIC algorithm. On one hand, we can only use shifters and adders for the multiplications with a constant multiplicand. The main idea of this method is decomposing the constant into a summation of several (−1)^*k*^2^*n*^ with different values of *k* and *n* and shifting the multiplier according to the values. *k* is 0 or 1, which determines the sign bit and the absolute value of *n* determines the number of bits that the multiplier need to be shifted. The direction of shifting is decided by the sign bit of *n*. If *n* is negative, the multiplier needs to be shifted to the left; if *n* is positive, the multiplier needs to be shifted to the right. On the other hand, the rest of multiplications are realized with functional shift multipliers (FSMs) with the structure shown in Figure 2. As we can see, one of the variables is split into single bits through a bus splitter and output to the multiplexers as enable signals. That is, if a bit of this variable is 0, the output of the corresponding multiplexer is 0; if a bit of this variable is 1, the output of the corresponding multiplexer will be the value of the other variable after shifting. The number of shifters is related to the bit position of the previous variable. Finally, add all the values from multiplexers and then the multiplication result can be obtained. It is worth noting that there is always one slow variable in a multiplication and splitting this variable is a better choice when using FSMs. Due to each additional bit of an FSM consumes a shifter and a multiplexer, all the FSMs used in this work are designed to fit the inputs in order to minimize the use of logical resources. The overall bit width is between 13 and 18.

Besides, when implementing the exponential operations with the CORDIC algorithm, we find that the iterative structure of *X* and *Y* is very similar, so we merge the two iterations and then the iterative Equation (4) becomes like Equation (9), which can save about one-third of the logical resources without changing the results. Under the premise of ensuring accuracy, the iterations of CORDIC is set to 20 for division and 10 for exponential operations in this work.

(9) $$\{\begin{array}{c} {\text{X}}_{\text{i+1}}={\text{X}}_{\text{i}}{\text{+q}}_{\text{i}}{\text{X}}_{\text{i}}\cdot {\text{2}}^{-\text{i}}\\ {\text{Z}}_{\text{i+1}}={\text{Z}}_{\text{i}}{-\text{q}}_{\text{i}}\cdot \tanh^{-\text{1}}\left({\text{2}}^{-\text{i}}\right) \end{array}$$

**Figure 3:**
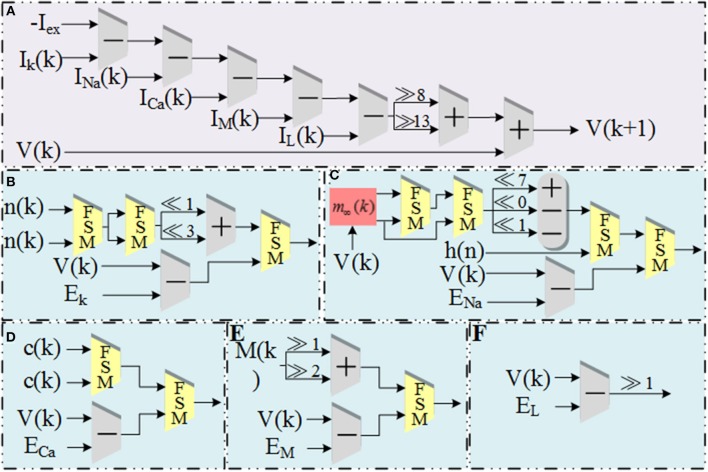
The schematic diagram of data flow for V and currents in the modified model. **(A)** The pipeline of “*V*.” **(B)** The pipeline of “*I*_*k*._” **(C)** The pipeline of “*I*_*Na*_.” **(D)** The pipeline of “*I*_*Ca*_.” **(E)** The pipeline of “*I*_*M*_.” **(F)** The pipeline of “*I*_*L*_.”.

**Figure 4:**
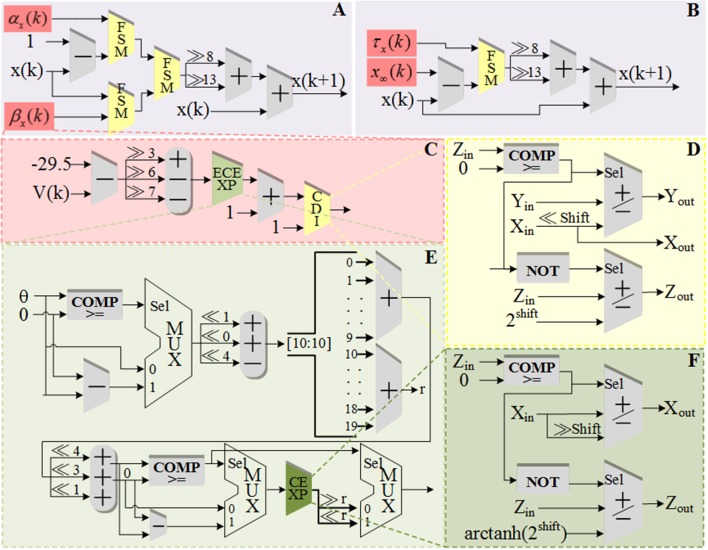
The schematic diagram of data flow for gating variables, a state variable and the detailed structures of one iteration in each CORDIC operations. **(A)** The pipeline of “*c*” and “*M*.” **(B)** The pipeline of “*h*” and “*n*.” **(C)** The pipeline of “*h*_∞_.” **(D)** One iteration of CORDIC division (CDI). **(E)** One iteration of expand CORDIC exponential (ECEXP). **(F)** One iteration of and CORDIC exponential (CEXP) in order.

The schematic diagram of dataflow for *V* is shown in Figure 3. Figure 3A shows the data flow of *V* with ionic currents and external current in the modified model. The detailed structures for the ionic currents are shown in Figures 3B–F. The schematic diagrams for the other four variables and the CORDIC algorithm are shown in Figure 4. Figure 4A is the structure for *c* and *M* and Figure 4B is for *h* and *n*. Due to space limitations, we only give a typical example for CORDIC-based non-linear function that includes all the compartments used in other functions in the Figure 4C. Figures 4D–F show the detailed structure for non-linear operations realized with the CORDIC algorithm. For the sake of simplicity, the figures only give the structure for one iteration of each operation and it will need several same structures with different values of “shift” for realizing the calculation. There is no LUTs and multipliers in all the designs so we can get a non-multiplier and non-LUT implementation through this method, which has potential for large-scale cerebellum realization on LaCSNN.

## Results

### Comparison of Software Simulation Results

**Figure 5:**
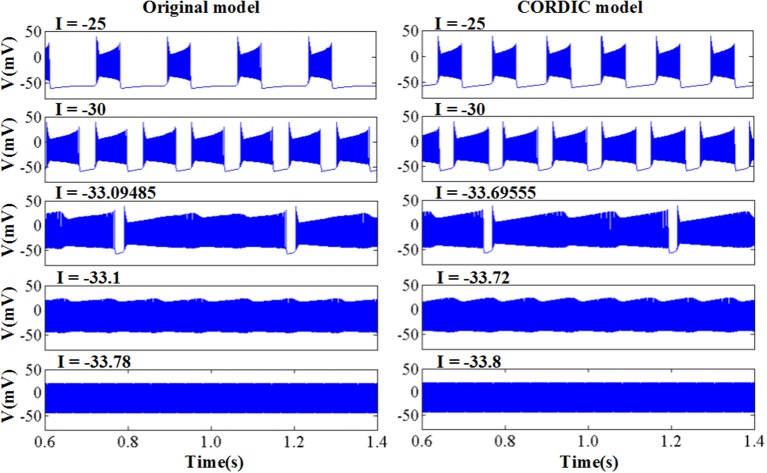
The voltage activities of original model and the modified CORDIC model.

**Figure 6:**
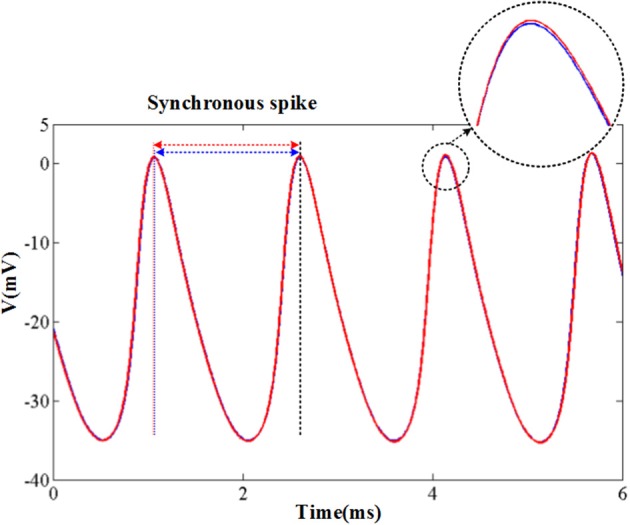
The spikes under fast spiking mode of the two models. The blue lines and red lines are for original model and the CORDIC model, respectively. The solid lines represent the spike waveform. The dash lines show the spike moment and the black one shows the synchronous spike moment of the two models. Horizontal arrows represent spiking intervals.

The original and CORDIC-based cerebellar Purkinje model are both simulated with MATLAB v2014a. The time step for software simulation is 0.004 ms. The membrane potential waveforms of two models are shown in Figure 5. As shown in the figure and taking the original model as an example, the burst activity with increasing amplitude can be seen when *I* = −25 (Bursting I). With the decrease of *I*, the interburst intervals decrease and the bursting becomes more durable (Bursting II). When *I* = −33.09485 (*I*′), a value in the critical region, the voltage activity presents bursts interspersed with amplitude modulation, which is the new type of activity founded in Kramer et al. (2008). The continuous decrease of *I* will make bursting disappear gradually, from the only spiking amplitude modulation (*I* = −33.1) to the complete fast spiking (*I* < −33.2). The CORDIC model can successfully reproduce the same voltage activities as performed in the original model but with different values of *I*. That is caused by the differences of the non-linear function realized with two different methods. There are inevitable errors of the CORDIC algorithm due to the iterative operations, but it will not affect overall results and can meet our requirements. To show it clearly, the detailed spiking waveforms for fast spiking of the two models are shown in Figure 6. We can see that there exists a certain but small difference in spiking interval and the disparity of amplitude is also limited.

### Error Analysis

In order to evaluate the CORDIC model more accurately, we use different methods to quantify the error between the two models to get a more comprehensive understanding of the CORDIC model. The detailed description of the method is as follows.

1) Maximum absolute error (mAE):

The absolute error (AE) is defined as the difference between the absolute values of the voltage of the two models. The maximum absolute error is defined as the difference between the voltage maximum absolute values of the two models. The two indexes can be calculated with the following equation:

(10) $$\{\begin{array}{l} \text{AE}\left(\text{i}\right)=\left|{\text{F}}_{\text{ori}}\left(\text{i}\right)-{\text{F}}_{\text{CORDIC}}\left(\text{i}\right)\right|\;,\;i=1,2,\ldots ,N\\ \text{ mAE}=\max\left(\left|F_{\text{ori}}\left(\text{i}\right)-F_{\text{CORDIC}}\left(\text{i}\right)\right|\right)\;,i=1,2,\ldots ,N \end{array}$$

where *F*_ori_(*i*) represents the relevant value of the original model and the *F*_CORDIC_(*i*) represents the relevant value of the CORDIC model. The symbol |·| is used to get the absolute value and the max(·) is used to get the maximum value. In order to get the error of the CORDIC algorithm in detail, we calculated the mAE of all nine non-linear functions realized by the CORDIC algorithm under a complete spiking waveform for a more convincing effect. The values are summarized in Table 2.

2) Root mean square error (RMSE):

The root mean square error is a typical measurement index for two value differences and very sensitive to very large or very small errors. We also calculate the RMSEs of all nine non-linear functions with the equation below and summarized in Table 2.

(11) $$\text{RSME}=\sqrt{\frac{1}{N}\sum_{i-1}^{N}({\text{F}}_{\text{ori}}(\text{i})-{\text{F}}_{\text{CORDIC}}(\text{i}){)}^{2}}$$

3) Error of spikes' timing (ERRt):

The error of spikes' timing reflects the difference in spiking interval between the two models. It can not only directly reflect the difference in spiking periodicity but also indirectly reflect the difference in the shape of the spiking waveform. To calculate the spiking interval, we should find a synchronous spike at first just as Figure 5. Then, measure the time interval between the synchronous spike and the previous or next spike. The error can be calculated as follows:

(12) $$\{\begin{array}{c} \text{ERRt}=-|\frac{\Delta {\text{T}}_{\text{CORDIC}}-\Delta {\text{T}}_{\text{ori}}}{\Delta {\text{T}}_{\text{ori}}}|\\ \Delta \text{T}={\text{t}}_{\text{syn}}-{\text{t}}_{\text{pre}}\text{ or }\Delta \text{T}={\text{t}}_{\text{nex}}-{\text{t}}_{\text{syn}} \end{array}$$

where Δ*T*_*CORDIC*_ represents the spiking time interval of the CORDIC model and the Δ*T*_*ori*_ represents the spiking time interval of the original model.

4) Correlation coefficient (Corr):

The correlation coefficient is an amount of linear correlation between the two groups of data. For the spiking waveforms of the two kinds of neurons, the larger the correlation coefficient is, the more similar the two waveforms are, and the maximum value of Corr is 1. As shown in Equation (13), the Corr is generally defined as the ratio of covariance to variance product of two sets of data. The covariance and the variance of the two sets of data can be obtained by Equation (14).

(13) $$\begin{array}{r} Corr=\frac{\text{cov}\left({\text{V}}_{\text{ori}},{\text{V}}_{\text{CORDIC}}\right)}{\sigma \left({\text{V}}_{\text{ori}}\right)\sigma \left({\text{V}}_{\text{CORDIC}}\right)} \end{array}$$

(14) $$\{\begin{array}{l} \text{cov}\left({\text{V}}_{\text{ori}},{\text{V}}_{\text{CORDIC}}\right)\\ =\sum_{\text{i}=1}^{\text{n}}\left({\text{V}}_{\text{ori}}\left(i\right)-\bar{{\text{V}}_{\text{ori}}}\right)\left({\text{V}}_{\text{CORDIC}}\left(\text{i}\right)-\bar{{\text{V}}_{\text{CORDIC}}}\right)\\ \sigma (\text{V})=\sqrt{\sum_{\text{i}-1}^{\text{n}}{\left(\text{V}(\text{i})-\bar{\text{V}}\right)}^{2}} \end{array}$$

**Table 2:** The value of RMSE and mAE of non-linear function realized with CORDIC.

**CORDIC functions**	**RMSE**	**mAE**
*n*_∞_	0.0013	0.0098
τ_*n*_	0.0010	0.0068
*h*_∞_	8.80 × 10^−4^	0.0073
τ_*h*_	0.0012	0.0102
*m*_∞_	0.0013	0.0082
α_*c*_	0.0012	0.0146
β_*c*_	0.0015	0.0299
α_*M*_	1.42 × 10^−5^	1.28 × 10^−4^
β_*M*_	5.22 × 10^−5^	4.29 × 10^−4^

We calculate the ERRt at 20 different times and take the average as the final value and the Corr is calculated with the membrane voltage values 6 ms (about 3–4 complete spiking) after the start of spiking synchronization. Both the two indexes are measured with five spiking modes and the values are summarized in Table 3. It can be seen from the table that the ERRt of the five spiking modes are <0.005, which indicates that the difference between the spiking intervals is small. In addition, the Corr of the five discharge modes is also around 0.99, which indicates that the spiking waveform is very similar.

**Table 3:** The value of ERRt and Corr of five different types of spikes.

**Spike type**	**ERRt**	**Corr**
Bursting I	0.0011	0.9980
Bursting II	0.0006	0.9890
Bursting with amplitude modulation	0.0037	0.9835
Amplitude modulation	0.0041	0.9922
Fast spiking	0.0005	0.9817

**Figure 7:**
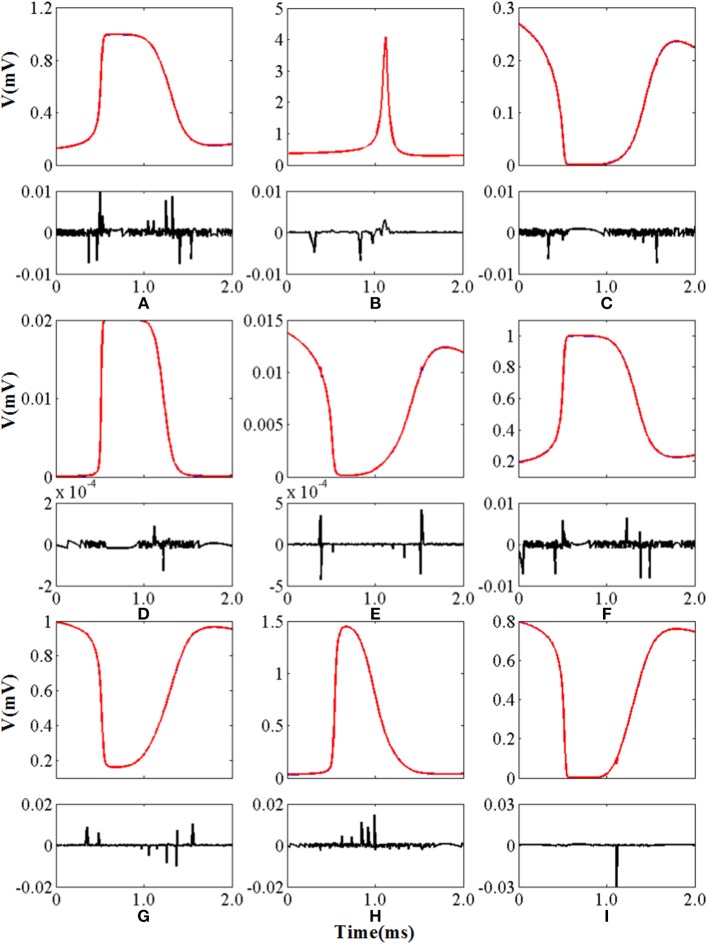
The curve and absolute error of nine nonlinear functions. The top panel of each figure is the function curve and the bottom panel of each figure is the absolute error. Blue lines represent the original nonlinear functions and red lines represent the CORDIC functions. **(A**–**I)** Represents *n*_∞_, τ_*n*_, *h*_∞_, τ_*h*_, α_*c*_, β_*c*_, α_*M*_, β_*M*_, and *m*_∞_ in order.

The curves of the nine non-linear functions of the original model and CORDIC model are shown in Figure 7 with the shape of AE below. As can be seen from Figure 7 and the two tables, the model implemented using the CORDIC algorithm has very small errors calculated by various methods and can meet the needs for building spiking neurons.

### Dynamic Analysis

In order to evaluate the difference between the two models more comprehensively, we learn and compare the dynamical mechanisms in different discharge modes. Because small errors can cause large differences in dynamical diagrams, it is a good way to measure model consistency. For more convincing, we have implemented the dynamic mechanisms in Kramer et al. (2008): the bifurcation diagrams of voltage *V* and the slow variable *M*.

**Figure 8:**
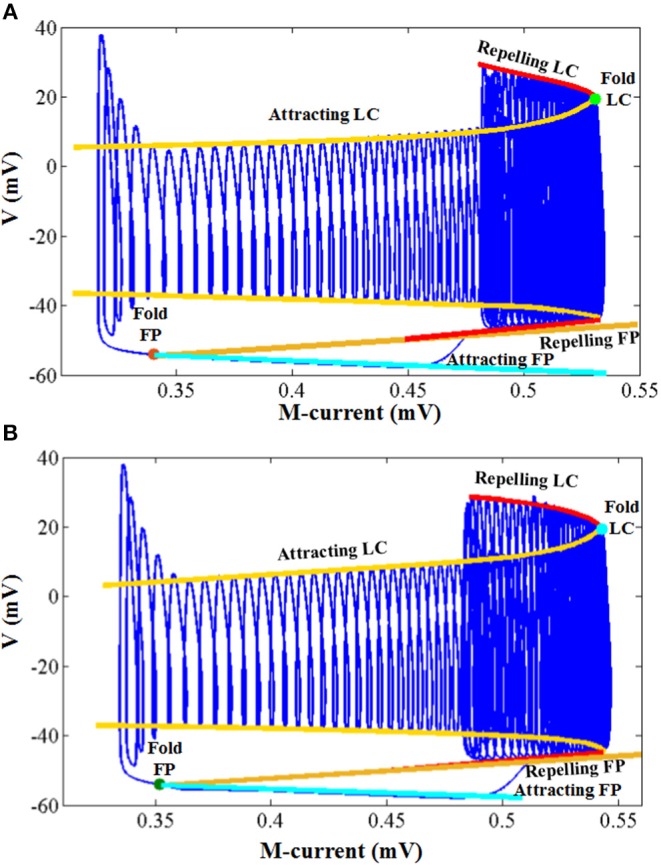
Comparison of the dynamics between the original model **(A)** and the CORDIC model **(B)** with *I* = *I*′. The “FP” and “LC” represent fixed points and limit cycles, respectively.

The simulation results of the entire system and its associated bifurcation diagram for bursting with amplitude modulation are shown in Figure 8A, which is obtained from the original model, and Figure 8B, which is from the CORDIC-based model. For a clearer description, the portion of the M-current at (0.483, 0.546) mV is referred to as the fast subsystem. When the M-current is reduced to less than the voltage at fold of fixed points in the fast subsystem, the rapid discharge begins and the attracting and repelling fixed points are also merged at this point. After that, the voltage increases rapidly and the system enters the fast subsystem along the attraction curve of the limit cycle. During this period, the M-current gradually increases until it reaches a fold of limit cycles in the fast subsystem. Finally, the M-current decreases, and the dynamics of the system temporarily follows the repelling branch of limit cycles until the return fixed points (light gray) or limit cycles. It can be seen from the figure that the small errors of CORDIC make the shapes of the two figures slightly different, but the CORDIC models can still reproduce the results in the original paper very well.

**Figure 9:**
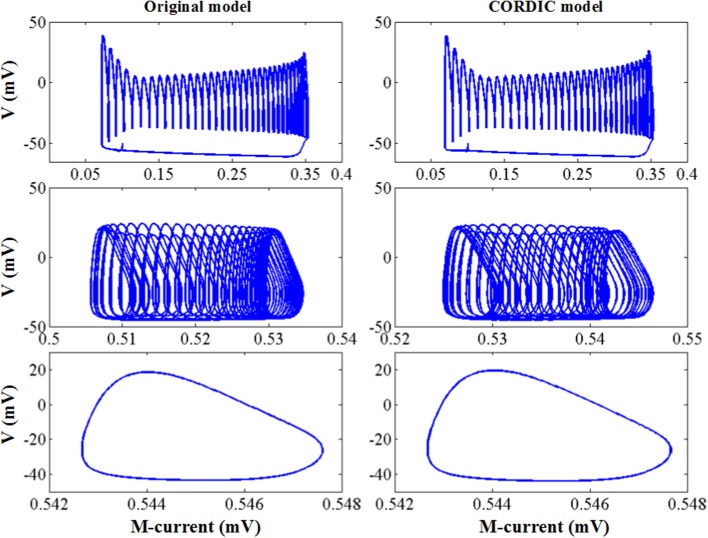
The dynamic of the original model **(left)** and CORDIC model **(right)** for bursting, amplitude modulation, and fast spiking.

Figure 9 shows the other three bifurcation diagrams for bursting, amplitude modulation, and fast spiking. M-current and calcium current play major roles in the switching of the spiking mode. When the hyper-polarization due to the M-current works (*I* < *I*′), the bursting occurs due to the victory of hyperpolarization, then the cell enters the stationary phase of bursting and spiking stops. When the calcium current works, its depolarizing effect prevents the hyperpolarization. Then, the stationary phase no longer appears, with the amplitude modulation spiking instead. As *I* continues to decrease, there only exists fast spiking. There are still small differences in these figures, but they are also able to reproduce the dynamical mechanisms that the cell follows.

### Network Behavior

In this section, we present a network of two coupled PCs to verify the proposed method. The two PCs (*V*_*pre*_ and *V*_*pos*__t_) are all in the form of Equation (1) each with an extra added synaptic current *I*_*syn*_. The pre-PC is set to an excitatory cell and the post-PC is set to an inhibitory cell. The pre one receives excitatory current *I*_*syn*_*post*_through GABA_A_ receptors and the post one receives inhibitory current *I*_*syn*_*pre*_ through AMPA receptors. The detailed synaptic current is shown as follows:

(15) $$\{\begin{array}{c} \frac{dz}{dt}=\frac{1+tanh(\frac{V}{10})}{2}\frac{1-z}{\tau_{1}}-\frac{z}{\tau_{2}}\\ I_{syn\_pre}=-2ptWz_{pre}(V_{post}-V_{inh})\\ I_{syn\_post}=Wz_{post}(V_{pre}-V_{ex}) \end{array}$$

*W* is neuron connection weight, *z* is the synaptic activation variable, and τ_1_ and τ_2_ are time delay constants, which for GABA_A_ receptors are 0.5, 10 and for AMPA receptors are 0.2, 2, respectively. The other parameter values are: *W* = 0.5, *V*_*ex*_ = 0, *V*_*inh*_ = −50, *I* for pre and post cell is −25 and −34, respectively.

**Figure 10:**
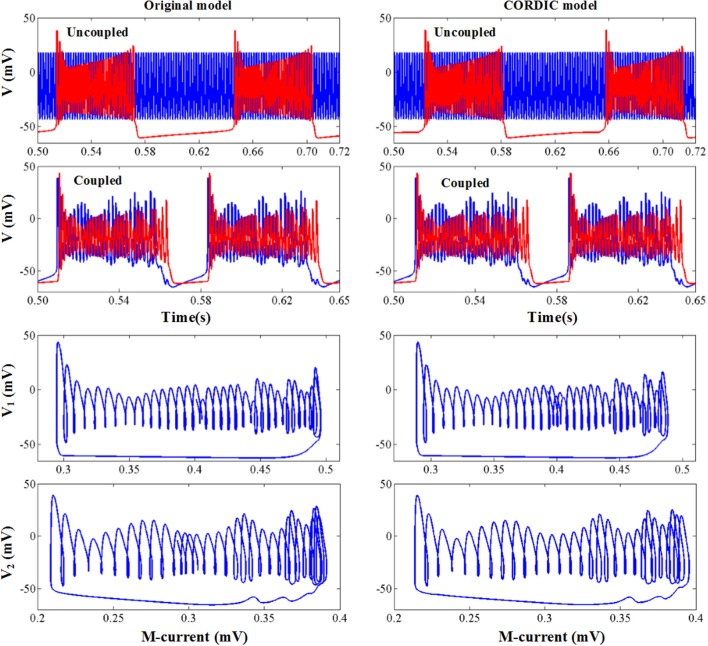
The network behavior of the original model **(left)** and the CORDIC model **(right)**.

The simulation results are shown in Figure 10. When the two neurons are uncoupled, they both present the spike mode according to the value of *I*, bursting mode for the pre cell with *I* = −25, and fast spiking mode for the post cell with *I* = −34. When the two neurons are coupled, the bursting period of the pre cell becomes longer due to the excitatory synaptic current and the spiking mode of the post cell turns into bursting due to the inhibitory synaptic current. It is worth mentioning that the peak value of each spike changes with time due to the interaction of the two neurons, and the dynamic behavior shows corresponding changes. We can see from Figure 10 that no matter the spiking behaviors or the dynamic behaviors, the original model and the CORDIC model show a high degree of consistency, which indicates that the proposed method is also applicable for the neural network.

### Hardware Implementation Result

**Figure 11:**
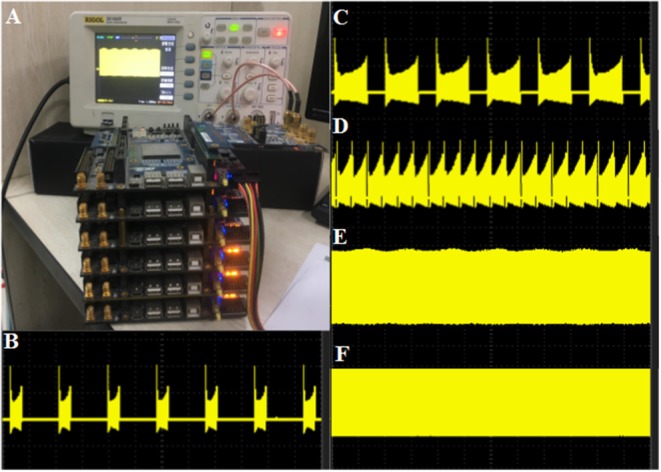
The hardware implementation results of the CORDIC cerebellar Purkinje cell on the LaCSNN system. The x-label is time with 100 ms/cell and the y-label is voltage with 50 mV/cell. **(A)** The LaCSNN system and the oscilloscope. **(B)** The membrane potential of “Bursting I” mode. **(C)** The membrane potential of “Bursting II” mode. **(D)** The membrane potential of “Bursting with amplitude modulation” mode. **(E)** The membrane potential of “Amplitude modulation” mode. **(F)** The membrane potential of “Fast spiking” mode.

The modified CORDIC cerebellar Purkinje model is built with the DSP Builder aided design toolbox in Simulink and then transformed to VHLD hardware language that can be compiled in Quartus II and downloaded to the LaCSNN system through USB-Blaster with Joint Test Action Group (JTAG) mode. In order to facilitate observation, the digital outputs from FPGA are transmitted to analog signals through a 16-bit dual-channel DA converter. The converter is also connected to an oscilloscope where the voltage activity of the model can be observed directly. The LaCSNN system and the voltage activity on the oscilloscope screen are both shown in Figure 11. The x-label and the y-label represent the time and voltage, respectively.

**Figure 12:**
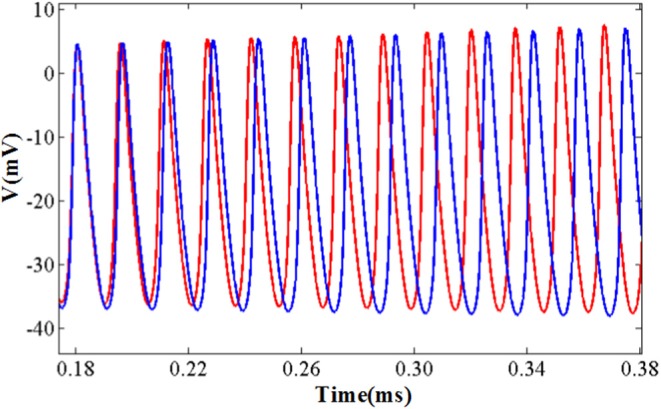
The detailed spiking shape of software simulation and hardware implementation under the fast spiking mode. The blue line represents the software simulation result and the red line represents the hardware implementation result.

**Figure 13:**
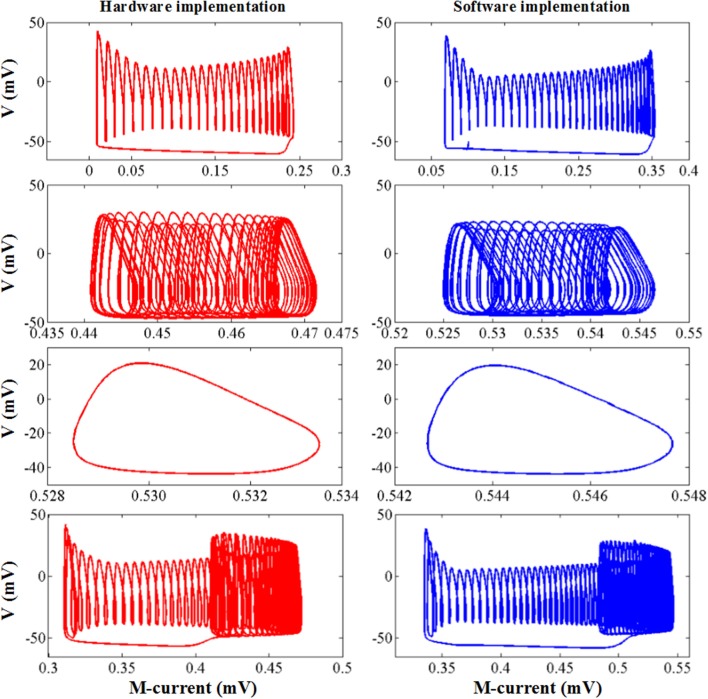
The dynamic behavior of software simulation and hardware implementation under bursting, amplitude modulation, fast spiking, and bursting with amplitude modulation mode. The blue line represents the software simulation results and the red line represents the hardware implementation results.

The comparison of the software simulation results and the FPGA implementation results for voltage activity is shown in Figure 12. To clearly present the difference between the two results, we give a partial spiking waveform. The overall shape of the voltage is the same, but the period and amplitude are different. The main reason is the usage of the approximation method and the fixed-point calculation on the hardware. The bifurcation diagrams for bursting, amplitude modulation, fast spiking, and bursting with amplitude modulation of the two simulation methods are shown in Figure 13; for the same reason, the basic shape of these diagrams is the same but the voltage values have deviations.

The resource utilization, working frequency, and power dissipation of the original and CORDIC model are summarized in Table 4. Due to the unroll iteration structure and a mass of multiplications, the logical elements used by the CORDIC model is more than the original model. However, the memory bits used by LUTs and the DSP block 18-bit elements used by multipliers can be reduced to zero. The power dissipation is a little more also due to the unroll iteration structure. For a clearer explanation, the same contents of the key algorithm of this method are summarized in Table 5. Comparing the three key algorithms, we can conclude that the FSM is more efficient than the CORDIC with less logic resources and high working frequency, which is why we do not use the CORDIC algorithm to realize multiplications. With the number of iteration increases (20 for division and 10 for exponent), the working frequency decreases due to the iterative structure, which affects the working frequency of the entire model. More importantly, it is obvious that there's no need for memory and multiplier resources for realizing the non-linear operations with high frequency and low power dissipation. It proves that, through this method, we can efficiently convert memory resources and multiplier resources into logical resources, which is of great significance to maximize the use of FPGA on-chip resources and improve the scale of neural network implementation.

**Table 4:** The resource utilization of hardware implementation for the two kinds of models on Altera Stratix III EP3SL340H1152C2.

**FPGA resources**	**Total available**	**Original model**	**Modified model**
Total logical elements	270,400	1,821	43,543
Dedicated logical registers	270,400	520	468
Total pins	744	29	29
Total memory bits	16,662,528	307,200	0
DSP block 18-bit elements	576	236	0
Total PLLs	4	1	1
Max frequency	–	28.15 MHz	53.44 MHz
Total power dissipation	–	275.40 mW	445.91 mW

**Table 5:** The resource utilization of hardware implementation for CORDIC algorithm and FSM on Altera Stratix III EP3SL340H1152C2.

**FPGA resources**	**Total available**	**CORDIC-Exponent**	**CORDIC-Division**	**14-bit FSM**
Total logical elements	270,400	644	1,222	303
Dedicated logical registers	270,400	565	537	0
Total pins	744	20	20	20
Total memory bits	16,662,528	0	0	0
DSP block 18-bit elements	576	0	0	0
Total PLLs	4	0	0	0
Max frequency	–	120.45 MHz	73.44 MHz	195.92 MHz
Total power dissipation	–	159.23 mW	164.81 mW	158.62 mW

## Discussion

There is a bottleneck for realizing a large-scale neural network with high biological precision neurons such as the model in this paper based on the H–H neuron model. These models have many conductance-based ionic currents that usually contain many non-linear functions and greatly increase the computational complexity. To solve this problem, many previous studies are working on FPGA resource optimization for spiking neurons with different methods (Ahmadi and Zwolinski, 2010; Bonabi et al., 2014; Hayati et al., 2016; Akbarzadeh-Sherbaf et al., 2018). Ahmadi and Zwolinski (2010) propose a method with PWL approximation for implementing the Izhikevich model. The non-linear operations in the model are only multiplications for there are no detailed ionic currents. The model complexity is relatively simple so the reference meaning for building high biological precision neurons is limited. Bonabi et al. (2014) implement an H–H-based model and a two-mini-column network with the CORDIC algorithm but it is only used for calculating exponent operations, but there are still some things to do to implement a large-scale neural network, because the multiplication and division operations account for a large proportion of the model and they still need multipliers and memory resources. Besides, there is no simplification for the iterative structure as we have done. Akbarzadeh-Sherbaf et al. (2018) use a general PWL approach to implement a randomly connected network with H–H models. If we just focus on one H–H model, the PWL approach can successfully realize the non-linear functions and improve the working frequency, but the precision is lower than the CORDIC algorithm for a sharp curve will certainly appear at the junction of the two linear sections. Besides, the approximate range of each linear part is only applicable to that set by the designer, so the linearization must be redesigned each time the model changes, and any unexpected values may get unexpected behaviors. As for the GPU platform, there may not be many researches on implementing a single neuron on it, but many researches have been carried on for the comparison between GPUs and FPGAs about implementing spiking neural networks (Cheung et al., 2012, 2016; Luo et al., 2016). The results show that GPUs can speed up the simulations with multi-core processors and parallel computing capacity, but compared to FPGA, two obvious cons still exist. One is the small on-chip memory and bandwidth, which limits the scale, the other is the high-power consumption of the desktop system. Besides, the calculation speed of GPUs is also lower than FPGAs in these works.

In order to save multiplier resources on FPGA, many multiplier-less methods have been proposed with different application ranges. Both Jokar and Soleimani (2017) and Hayati et al. (2016) propose a multiplier-less structure with the PWL approach that needs to linearize each function that contains multiplication of variables. The multiplier-less implementation in Agostini et al. (2005) and Gomar and Ahmadi (2014) are simple for there are all constant number multiplications in their models, which can be easily replaced by adders and shifters. Thomas and Luk (2013) replace the multipliers with LUTs and block RAMs, which use more LUT resources to save multiplier resources. Our work presents an FSM, which is common to all multiplication operations and easy to use. With this method, users do not need to redesign the whole approximation using the PWL approach, and all of the multiplications can be realized just by adjusting the supported bit width, even simpler than the method implementing the constant number multiplications. The working frequency of the FSM is 195.92 MHz as shown in Table 5, so the lower working frequency of the cell model compared to the model mentioned above is only due to the unavoidable iterative structure of the CORDIC algorithm and the complexity of this model.

This paper presents a multiplier-less and LUT-less CORDIC method to realize the conductance-based cerebellar Purkinje model on FPGA. This can be used for the trade-off among logic resources, memory resources, and multiplier resources, which can be adopted to make full use of the FPGA resources to build a large-scale neural network. All of the calculation modules in our work, the FSM, CDI, and ECEXP, can be directly used for any other models without any extra operation. Besides, the modified pipelined parallel CORDIC algorithm can significantly reduce the resource consumption and the complexity of the hardware implementation architecture.

## Conclusion

In this work, we present an efficient implementation of a modified cerebellar PC using the CORDIC algorithm with recently found new dynamic performance. Through the analysis of various errors of the two single-neuron models and the comparison of waveforms and network behaviors from different aspects, it can be concluded that the original model and the CORDIC-based model are consistent in biological activities and dynamic mechanisms. After that, we use the non-multiplier and non-LUT methods and implement the CORDIC model on the LaCSNN system. The implementation results are observed on the oscilloscope through the DA conversion module, which are also consistent with the results of the software simulation. By comparing the resource utilization of the original model and the CORDIC model in FPGA implementation, we can conclude that the method used in this paper can transform the use of multiplier resources and memory resources into logical resources, so as to maximize the utilization of FPGA on-chip resources and expand the network scale that can be achieved. This work provides an effective method for realizing large-scale spiking neural networks of cerebellum or many other spiking neural networks on FPGAs.

## Data Availability Statement

All datasets generated for this study are included in the manuscript/supplementary files.

## Author Contributions

XH, SY, XW, JW, BD, and GY made significant contributions to the conception and design of manuscripts and the drafting of manuscripts, as well as critical changes to important intellectual content. All authors have approved publications in their current form. All authors agree to be responsible for all aspects of the work to ensure proper investigation and resolution of issues related to the accuracy or completeness of any part of the work.

### Conflict of Interest

The authors declare that the research was conducted in the absence of any commercial or financial relationships that could be construed as a potential conflict of interest.
